# Optimising refugee children’s health/wellbeing in preparation for primary and secondary school: a qualitative inquiry

**DOI:** 10.1186/s12889-019-7183-5

**Published:** 2019-06-27

**Authors:** Jess R. Baker, Shanti Raman, Jane Kohlhoff, Ajesh George, Catherine Kaplun, Ann Dadich, Catherine T. Best, Amit Arora, Karen Zwi, Virginia Schmied, Valsamma Eapen

**Affiliations:** 1 the University of New South Wales, Liverpool Hospital Mental Health Centre Level 1, Liverpool, NSW 2170 Australia; 2 South Western Sydney Local Health District, Health Services Building Level 3, Cnr Campbell & Goulburn St, Liverpool, NSW 2170 Australia; 3 School of Psychiatry, University of New South Wales, Hospital Rd, Randwick, NSW 2031 Australia; 4 Karitane, 138-150 The Horsley Dr, Carramar, NSW 2163 Australia; 5 Centre for Oral Health Outcomes & Research Translation (COHORT), Western Sydney University, Locked Bag 7103, Liverpool BC, NSW 1871 Australia; 6 South Western Sydney Local Health District, Locked Bag 7103, Liverpool BC, NSW 1871 Australia; 7 University of Sydney, Locked Bag 7103, Liverpool BC, NSW 1871 Australia; 8 Ingham Institute for Applied Medical Research, Locked Bag 7103, Liverpool BC, NSW 1871 Australia; 9 Western Sydney University, Locked Bag 1797, Penrith, NSW 2751 Australia; 10 Ingham Institute for Applied Medical Research, Locked Bag 1797, Penrith, NSW 2751 Australia; 11 School of Business, Western Sydney University, Locked Bag 1797, Penrith, 2751 Australia; 12 Western Sydney University, The MARCS Institute, Locked Bag 1797, Penrith, NSW 2751 Australia; 13 School of Science and Health, Penrith, NSW 2751 Australia; 14 Translational Health Research Institute, Western Sydney University, Penrith, NSW 2751 Australia; 15 Discipline of Child and Adolescent Health, Sydney Medical School, Faculty of Medicine and Health, The University of Sydney, Westmead, NSW 2145 Australia; 16 Oral Health Service, Sydney Local Health District and Sydney Dental Hospital, NSW Health, Surry Hills, NSW 2010 Australia; 17 Sydney Children’s Hospital, Corner Avoca and Barker Street, Randwick, NSW 2031 Australia; 18 Academic Unit of Child Psychiatry South West Sydney (AUCS), University of New South Wales & Ingham Institute, Elizabeth Street, Liverpool, Sydney, 2170 Australia; 19 Liverpool Hospital, Elizabeth Street, Liverpool, Sydney, 2170 Australia

**Keywords:** Refugee, Health service, Preschool, Adolescent, Qualitative

## Abstract

**Background:**

Children from refugee backgrounds are less likely to access appropriate health and social care than non-refugee children. Our aim was to identify refugee children’s health/wellbeing strengths and needs, and the barriers and enablers to accessing services while preparing for primary and secondary school, in a low socio-economic multicultural community in Australia.

**Method:**

Ten focus groups were facilitated with Arabic-speaking refugee parents of children aged 2–5 years (*n* = 11) or in first year secondary school (*n* = 22); refugee adolescents starting high school (*n* = 16); and key service providers to refugee families (*n* = 27). Vignettes about a healthy child and a child with difficulties guided the discussions. Data was thematically analysed and feedback sought from the community via the World Café method.

**Results:**

Personal resilience and strong family systems were identified as strengths. Mental health was identified as a complex primary need; and whilst refugees were aware of available services, there were issues in knowing how to access them. Opportunities for play/socialisation were recognised as unmet adolescent needs. Adults spoke of a need to support integration of “old” and “new” cultural values. Parents identified community as facilitating health knowledge transfer for new arrivals; whilst stakeholders saw this as a barrier when systems change. Most parents had not heard of early childhood services, and reported difficulty accessing child healthcare. Preschooler parents identified the family “GP” as the main source of health support; whilst parents of adolescents valued their child’s school. Health communication in written (not spoken) English was a significant roadblock. Differences in refugee family and service provider perceptions were also evident.

**Conclusions:**

Refugee families face challenges to accessing services, but also have strengths that enable them to optimise their children’s wellbeing. Culturally-tailored models of care embedded within GP services and school systems may assist improved healthcare for refugee families.

**Electronic supplementary material:**

The online version of this article (10.1186/s12889-019-7183-5) contains supplementary material, which is available to authorized users.

## Background

At the end of 2017, global refugee numbers were at a record high, of which children made up 52% [1]. The number of refugees resettled in 2017 (predominantly in high income countries) was 102,800; and relevant to the setting of this paper, 15,100 of those persons were resettled in Australia [1]. Resettled refugee families arrive in their “host” country with similar health problems to their native-born counterparts, as well as issues specific to their birth country and migration experience [2]. Trauma affects development and family functioning, and interrupted schooling is common, thus additional support related to developmental and learning needs could be required. Yet, despite elevated rates of mental health symptoms and disability among refugee children, [3, 4] engagement with child health services is underutilised and tenuous [2, 5–9]. Such health inequities are unjust, systematic, and importantly, modifiable [10]. They are at odds with international mandates that refugees have the same fundamental right as all human beings to the enjoyment of the highest attainable standard of physical and mental health and equitable access to refugee-sensitive health services [11]. The number of refugees resettling in high-income countries, such as Australia, is growing. Research into addressing this inequity is essential and pressing.

Access to quality care during early childhood may be the most cost effective way to tackle inequity [10]; it can create a healthy “bedrock” or start to life for children and increase opportunities to deter the downward social gradient of disadvantage. Adolescence is also a pivotal time when access to quality healthcare is integral. It represents marked developmental growth, pubertal change and identity formation [12]. For adolescents from refugee backgrounds it can be an especially challenging time, as they negotiate multiple ethnic identities and may make new meaning from past trauma [13].

Most welcoming countries offer, in principle, some kind of medical screening for refugees (child and adult) upon arrival [14] . For example, the Australian state of New South Wales (NSW), offers a refugee health nurse screening program. Evaluations of the initiative indicated high levels of screening coverage. Specifically, nurses based in community clinics completed a physical health screen on 97% of eligible newly arrived refugees aged 0–15 years [15], while those in Intensive English Study Centres (precursors to entering High School), screened 90% of students, of whom 80% were found to have two or more medical conditions [16].

Countries vary, however, in the quality of screening and comprehensive healthcare they provide and how well refugees ultimately benefit from it [17]. Legal restrictions and imposed waiting periods before refugees are eligible to access services can delay care. In Australia, health service entitlements can vary by visa category, whereby refugees who arrive on a “sponsored” visa may not access the screening program (and other services) due to expectations that their sponsor can support them [18]. Moreover, despite the high rates of trauma documented within refugee children/youth and the ensuing impacts on life success, many screening programs overlook mental health and social wellbeing [18, 19].

While the health assessment is a valuable starting point for eligible refugees, the pathway of care thereafter is often uncertain [17]. In the evaluation of the screening program based out of Australian Intensive English Study Centres, uptake of GP referrals was confirmed in only two-thirds of cases [15]. Furthermore, the ways in which children and their parents navigate higher-level specialist services or longer-term preventative healthcare is also not well understood. Frequently cited barriers to pursuing a referral include limited English language competency, inadequate interpreter provision, fear or distrust of services, and financial and transport challenges [2, 18, 20–25].

Even if such barriers are addressed, many refugee families do not identify or prioritise the same needs for their child as practitioners might. Management of resettlement stressors such as adequate food and housing for the family, are understandably often prioritised over personal health [22, 26]. This emphasises the importance of ensuring that refugee families are essential partners in the development of models of care for their children [27], as well as both health and non-health resettlement professionals [28, 29]. In summary, our study aimed to identify (i) the strengths and needs of refugee pre-schoolers and adolescents in anticipation of the critical transitions into primary or secondary school respectively and; (ii) the barriers and enablers to their engagement with quality childhood healthcare, from the perspective of refugee families and the health and non-health resettlement professionals who provide key services to them.

## Methods

### Design

The design was a qualitative study. The study was approved by the South Western Sydney Local Health District Human Research Ethics Committee (reference number: HREC/17/LPOOL/269).

### Setting

The research took place in a low socio-economic multicultural community in Sydney, Australia. This community welcomes a significant minority of Australia’s refugee intake each year. Between 2010 and 2015, for example, over 12% of Australia’s 73,833 refugee intake was settled here; an additional 12,000 Syrians were also resettled in the area between 2016 and 2017 [30].

### Participants

#### Parents and adolescents

Parents who had previously participated in a university study, and provided written permission to be contacted about future research activities, were invited to participate. The original sample was recruited from within five local primary schools. Specifically, all children in Year 5 and 6 at school (ages 9–12 years), who had arrived on an Humanitarian visa were invited by a school staff member to participate in a longitudinal quantitative study examining how school climate relates to the mental health of refugee students in the transition from primary to secondary school [Baker, Silove, Horswood, Al-Shammari & Eapen: How School Climate Relates to the Wellbeing and Resettlement of Refugee Children, in preparation]. The response rate in the original sample was 93% (233 out of an eligible 250 participants). The Arabic-speaking researchers who met with the families from the original sample, were the same researchers who facilitated the present study’s focus groups, thus there was an established relationship.

Parents were eligible to participate if they spoke Arabic, had arrived in Australia on a humanitarian visa at least 3 months prior, and had either a child aged 2–5 years or a child that had recently transitioned into the first year of secondary school (aged 12–13 years). The adolescent children of the participating parents were also invited to participate. There were no unaccompanied minors in the sample.

Participant information and consent forms were translated into Arabic. The bilingual research team read through the form with participants and confirmed their comprehension. All parents and adolescents gave written consent prior to participation, with adolescents providing consent using a simplified, age-appropriate consent form. Parent and adolescent participants were reimbursed for their time with $30 and $10 gift vouchers, respectively.

#### Stakeholders

A study advertisement was circulated to organisations that engage with refugee families in the local community. Stakeholders were eligible to participate if they had worked at an organisation that engages with refugee families for at least 6 months. All stakeholders provided written consent.

### Procedure

#### Data collection

Ten focus groups were conducted (two involving preschool parents; three involving adolescent parents; two involving adolescents; three involving stakeholders). Four hypothetical vignettes of a refugee child and their family guided the focus group discussions. Two vignettes described a healthy developing adolescent or an adolescent with developmental or socio-emotional issues; and two vignettes described preschool-aged equivalents (see the Focus Group Guide in the Files). Some of the issues described included intellectual, physical and sensory impairments, as well as oral health, mental health, and behavioural issues and peer difficulties, within the context of an absent or unwell parent.

The vignettes were developed by a small steering committee comprising of clinicians, academics and policy makers who are involved in the provision of health services to local refugees. The vignettes underwent several iterations until all committee members agreed that the content was appropriate and typical of refugee children and families who present to local health services. Preschool parents were presented with the two pre-schooler vignettes. The adolescents and adolescent parents were presented with the two adolescent vignettes. Stakeholders were presented with one vignette of a pre-schooler and one vignette of an adolescent; one being healthy and one with health issues - the chosen vignettes were counterbalanced across the three stakeholder groups.

Parent focus groups were facilitated in Arabic (with vignettes and questions verbally translated) by female bilingual research assistants. Using a strength/needs-analysis approach, the focus group questions sought to ascertain parents’ challenges, needs, strengths, access and perception about health services in order to assist a successful transition into primary or secondary school for their child (see the Focus Group Guide in the Additional file 1). The adolescent focus groups were conducted in English and facilitated by the first author and a bilingual assistant. They pursued a similar line of inquiry to the parent groups, with stronger emphasis on personal and social resources and needs, and sourcing additional support more broadly rather than experiences with health services per se.

Stakeholder groups were conducted in English and facilitated by the first author and a bilingual assistant. They explored perspectives on access and quality of healthcare for refugee children, in the context of the current health systems and collaboration. Focus groups were digitally recorded and field notes also taken. A professional company transcribed the English audio recordings verbatim. The recordings from the focus groups conducted in Arabic were transcribed in Arabic and then translated into English by the bilingual research assistants who co-facilitated the focus groups. Demographic data was also obtained from participants. Parent and adolescent demographic data was obtained via a translated self-report with the aid of the Arabic-speaking focus group facilitators.

#### Data analysis

Thematic analysis was initially largely inductive [31]. Following an independent appraisal of the data, six members of the research team convened to identify and compare common patterns and lower order themes and collaboratively develop a tentative coding framework. The first and second authors then returned to the transcripts and refined and revised the code definitions and grouped similar codes into higher order themes as they emerged. The iterative process became more deductive as the analysis progressed in identifying refugee children’s health and wellbeing strengths and needs, as well as the barriers and enablers to accessing health and support services at the primary and secondary school transitions [32]. A final coding framework was developed and a subset of transcripts coded again by independent team members (including a second meeting) until consensus in reliability of codes and overall themes was achieved. The first author then returned to the full set of transcripts to confirm that the thematic analysis was representative of the full dataset, in that all ideas were signified.

Triangulation of the four populations assisted in enhancing data reliability and saturation [33]. Saturation appeared to be achieved at the data level in that no relevant new insights were emerging from the third adolescent and stakeholder focus groups; and at the thematic level, in that the same themes but no new relevant ideas were occurring [34].

#### World Café

To maximise study rigour, the process was guided by Guba’s four criteria for trustworthiness [35]. This included inviting community feedback on the study findings via a world café method [36–38]. Specifically, 35 participants representing 17 different organisations that support the local refugee community (representing sectors such as disability, education, refugee health, oral health, paediatrics, child psychiatry, early childhood, pre/peri/post-natal maternal health, and English learning centres), as well as expert academics and parents from a humanitarian background, were invited to participate in a structured discussion of the draft themes in an informal setting.

Specifically, world café participants were invited to discuss what surprised them about the study findings; and what resonated with them. Participants then brainstormed ideas to facilitate better access to health services for refugee children in preparation for school; and considered the importance and ease of the ideas via allocation of each idea into one of four boxes (see Table 1). A nominated scribe recorded the breakaway group discussions, which were collated and summarised by the first author. No demographic data were collected on the attendees.

**Table 1:** Adapted PRECEDE-PROCEED implementation matrix [39]

	More Important	Less Important
Easy to Do	High priority	Low priority
Harder to Do	Innovation priority	No priority

## Results

### Participant demographics

The final refugee sample consisted of 11 parents of preschool-aged children, 22 parents of adolescents, and 16 adolescents (see Table 2). The majority of families were Arabic-speaking and from Syria and Iraq. Parents were predominantly mothers, and duration lived in Australia was broad. The final stakeholder sample consisted of 27 participants across 17 different organisations representing child and family health services, oral health, paediatrics, psychiatry, counselling, community services, settlement services, education departments, and government benefit and housing providers. The majority were female (*n* = 25) and all were educated at a diploma level or higher. Stakeholders had worked with their current employer for an average 4.6 years (range 0.2–17 years) and 21 stakeholders reported training or qualifications in working with people from refugee backgrounds. Five stakeholder participants spoke Arabic in addition to English.

**Table 2:** Parent and Adolescent demographics

Demographic Variables	Preschool parents (*n* = 11)	Adolescent parents (*n* = 22)	Adolescents (*n* = 16)
Gender: Female (n; %)	11 (100)	17 (77.3)	9 (56.3)
Age in years (M (SD);	37.77 (6.24)	44.55 (6.70)	12.56 (.73)
Duration in Australia (months) (M (SD);	35.10 (35.24)	49.18 (33.37)	40.00 (22.53)
Current Visa			
Refugee/Humanitarian	8 (72.8)	17 (77.2)	16 (100)
Permanent Resident/Citizen	2 (18.2)	4 (18.2)	
Country of Birth (n; %)			
Iraq	11 (100)	19 (86.4)	12 (75.0)
Syria	0	3 (13.6)	4 (25.0)
Ethnicity (n; %)			
Arabic	0	3 (13.6)	
Assyrian	2 (18.2)	5 (22.7)	5 (31.3)
Chaldean	2 (18.2)	5 (22.7)	6 (37.5)
Iraqi	3 (27.3)	3 (13.6)	
Mandaean	4 (36.4)	5 (22.7)	5 (31.3)
Syrian	0	1 (4.5)	
Marital Status: married (n; %)	10 (90.9)	20 (90.9)	
Employment Status: Full-time parent/carer (n;%)	10 (90.0)	11 (50.0)	
Annual Household Income (n; %)			
Under $18,000	1 (9.1)	2 (9.1)	
$18,201 - $37,000	10 (90.9)	11 (50)	
$37,001 - $80,000	0	6 (27.3)	10 (62.5)
Highest Level of Schooling (n; %)			
No formal schooling	0	1 (4.5)	
Primary/secondary	7 (63.7)	12 (54.6)	
Diploma	2 (18.2)	4 (18.2)	
Bachelor Degree	2 (18.2)	3 (13.6)	
Language Spoken at Home (n;%)			
Arabic	7 (63.6%	11 (50.0)	6 (37.5)
Assyrian	4 (36.4)	6 (27.3)	4 (25.0)
Chaldean	0	5 (22.7)	4 (25.0)
Speak English? (n; %)			
Very well/Well	3 (27.3)	6 (27.3)	16 (100.0)
A little/ Not at all	8 (72.7)	15 (68.1)	
Understand spoken English? (n; %)			
Very well/Well	4 (36.4)	6 (27.2)	16 (100)
A little/Not at all	7 (63.6)	15 (68.1)	0

numbers that do not add up to 100% indicate missing data

### Focus group findings

**Fig. 1:**
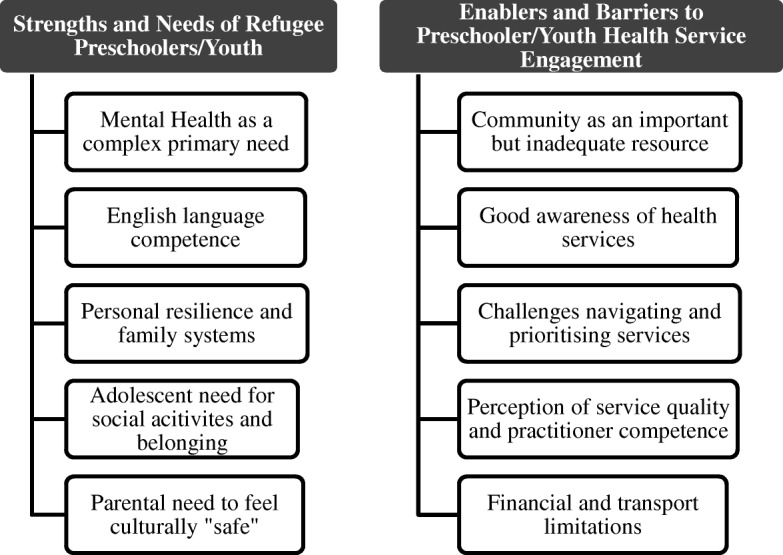
Summary of main thematic findings

Results were organised under two main themes - strengths and needs of refugee pre-schoolers and adolescents; and enablers and barriers to quality childhood service engagement. Within each sub-theme several lower-order themes were identified (see Fig. 1 for a summary).

### Strengths and needs of refugee preschoolers and adolescents

#### Mental health acknowledged as a complex primary need

Across participant groups, supporting mental health needs in order to facilitate successful learning at school was a standout theme. Moreover, parents and adolescents made quite normalising references to mental health.

*“The psychological condition is the most important thing for a person … I tell you about three countries, there is no one left without a psychological condition … what we’ve been through, our kids because of the bombing they are too scared. Flashbacks … He wants to learn, but he can’t concentrate.”* (Parent)

One adolescent girl suggested that it would be helpful to have “*brain surgery … [to] take the bad bit [memories] out”*. However, some implicit or subtle stigma was still apparent; for example, the parent continued, *“Even the one who visits the psychiatrist he doesn’t tell others that he has an appointment with the psychiatrist.”* Stakeholders illustrated this stigma with the belief verbalised by some of their clients that *“if you are a young adult, apparently you can’t get married if you’re going to [counselling]”*; and intimated that some externalising behaviour receives discrimination from the community, “*Especially if you have a child who’s a bit unruly. Some behaviour issues, the judgement around that, rumour, gossip”.*

#### Personal Resilience and Family Strengths

A dominant theme was of personal resilience. Parents and adolescents testified to being self-sufficient, hopeful, and holding a faith, as important values.

“*Any human who comes here, if he wants to adapt himself to this country, then he won’t need help to do so. He would realise the strengths in this country straight away … The family must encourage themselves, ask, find out, depend on themselves … the solutions are inside us!”* (Parent)

Stakeholders and parents alike spoke of healthy proactive parents, especially mothers, with “strong” personalities as being an asset in navigating services for their child. For example one parent said, “*self- confidence for the father and the mother, if they then implement self-confidence inside the child.”* Having siblings and extended family in Australia was also acknowledged as a significant strength. Conversely, stakeholders spoke of a need to address instability, lack of routine and permissive parenting in families.

#### Competence in English language

Participants collectively endorsed that stronger competency in English language (for both parents and adolescents) was a significant strength in terms of being able to communicate with child services, exercise independence, assist the child’s education and interact with the community. An adolescent recognised that *“[the child] has the language; it solves a lot of problems”*. A distinction was made between written and spoken English, in that all participants endorsed that it is substantially harder to learn written English; and that this is problematic as health service communication is often through letters. Parents reported “*throwing the [letters] out”* when they arrive, because they cannot read them. Government provision of English language lessons to many humanitarian entrants was identified as helpful in addressing this need. However, it was also highlighted that mandates for adults to attend a set number of English lessons per week can restrict parents’ availability to take their children to appointments, and that systems could potentially be improved by greater flexibility surrounding attendance. One stakeholder said: “*It’s actually physically impossible for people to get their kids to school, get to [English class], go back, and go to an appointment.”* Striking a balance between rapid learning of English language versus accommodating health appointments within the class timetable was also endorsed by World Café participants.

#### Adolescent need for social activities and belonging

Parents spoke of the importance of a prosocial peer group, and of play, socialisation, sports, and the pursuit of meaningful hobbies - for “joy” but also as a distraction from other issues. Echoing their parents, adolescents spoke of the importance of play and friendships to feel connected.

*“In Australia you don’t know what the hell is going on… She needs friends and stuff to socialise and communicate and have fun … maybe they can make a website that would tell them how to get more involved in social life and be part of the community.”* (Adolescent boy)

Stakeholders highlighted a gap in psychosocial services for the adolescent age group. They also recognised that having access to a Smartphone and the internet was important for the adolescent - although warned that typically uncensored social media “*all the videos that get put up about the bomb blasts that are going off in Syria and Iraq”* can be a traumatic trigger for adolescents.

#### Parental need to feel culturally ‘safe’

Stakeholders spoke of the most fundamental need for families to feel that their child is safe. Preschool parents voiced concerns that “*they [authorities] even take the kids from their families”* as a barrier to reporting any concerns or admitting that they are struggling. Adolescent parents reported a more abstract fear of losing their child to Australia, or the “bad” influence of non-prosocial Australian peer influences. One parent said, *“we have to teach her how to grow between … how to protect her culture and at the same time adapt to Australia because … the Aussies take her to a different world, to a different way … the Arabic services aren’t well.”* This was accompanied by sentiments of feeling unable to bring up their child the way they wish. Adolescents did not reference this idea explicitly but spoke of missing their home country.

Stakeholders described this as a need to provide health services that are sensitive, respectful and inclusive of dual cultures. This was corroborated by World Café participants, who detailed different cultural norms regarding what is acceptable in terms of child-raising practices; and the need for space to discuss this and provide reassurance within service provision. Associated concerns included the implications that this need or fear has, in regards to early intervention and parent-driven approaches to accessing care if parents are delaying to be seen.

### Enablers and barriers to quality childhood service engagement

#### Community as an important but possibly inadequate resource

Parents spoke of the community and previously settled refugee families as an invaluable knowledge resource for newly arrived families. One parent shared *“he who came before him will help him”*. An adolescent similarly said, *“We ask the people who have been here before us*”. However, stakeholders recognised that this as an issue when systems frequently change and families then hear outdated or confusing information, *“the blind leading the blind”.*

#### Good awareness of health services

Parents and adolescents named a substantial number of health and support services including mental health services, and spoke of using the internet to gather health information. This awareness of services surprised some World Café participants. However, refugee families were unaware of (free) early childhood centres that provide support and information on parenting issues for children aged 0–5 years. Stakeholders (and World Café participants) agreed that there was minimal refugee family attendance at playgroups, and elaborated that such services are often viewed as unnecessary if the family are connected with a GP. Parents of pre-schoolers spoke more about the GP being their go-to person for health support. One stakeholder described the GP as “*a very valued, highly respected member within their culture”*. Whilst adolescents recommended going to the school counsellor for help, their parents spoke more about the school as being their main support.

*“I really think the school is the main one to help in everything … it’s more important than any of these organisations … it guides, leads, teaches and educates … the school is the right and the best one to direct the child to those [services]. I have learned about [the services], through the school if it wasn’t for the school, I wouldn’t have known.”* (Adolescent parent)

#### Navigation and prioritisation of services

Whilst families evidenced an awareness of services, this was distinct from an ability to manage, navigate or prioritise all the named services. One parent said: “*I just miss the simplicity of life back home. I just miss how simple life was and in Australia you have to be on top of everything all the time”*. A central person whom families could trust was identified as critical in managing all the services and appointments. Linking services and the importance of a case manager in meeting refugee youths’ needs was reiterated by World Café participants. Stakeholders also identified a need to stage or prioritise services, and importantly, to reconcile the disconnect that sometimes occurs between families’ and practitioners’ priorities. For example, one practitioner identified addressing domestic violence within the home as a possible priority in contrast to the parents identifying keeping the family together as a priority.

*“Everyone wants to help … they’ve got so many providers going into their home, often they’ve no idea who anybody is. We don’t ask them to prioritise their needs. We assume, oh, they’ve got five things wrong, we need to help with five things. And sometimes they’ll go to something that really, they could have gone to six months later, and they missed their dental appointment which is most important right now.”* (Stakeholder)

#### Perception of service quality and practitioner competence

Parents’ overwhelming gratitude and predominantly positive view of services was a ubiquitous theme. One parent shared, *“This county loves to help … We say, ‘Thank God’ a thousand times for everything they are giving us”.* This contrasted with stakeholder perspectives, which were much less positive about services for refugee families. They queried whether families “*don’t know what they don’t know”*, and questioned whether their sense of gratitude prevents them from articulating their concerns and needs.

*“They’ve gone for a specialist appointment they’ve waited 3 months for and had some hiccup with the translator … They say, thank you very much I’ll wait again, but the truth is they’re absolutely devastated, they’re in pain and they couldn’t express that they were in pain.”* (Stakeholder)

Some World Café participants conveyed surprise about refugee parents’ reported gratefulness for services, whilst others reported encountering similar sentiments of refugee families “taking what they are given”.

Stakeholders (and World Café participants) spoke of subtle discriminations among practitioners towards clients and wondered how knowledgeable some were in supporting refugee families. For example, “*The GPs you see they’re trying to do their best, but they’re confused because they don’t have the knowledge to manage these children … because there’s very complex medical problems,”* said one practitioner. Some participating stakeholders said they did not feel fully confident in supporting refugee clients. They also spoke of inappropriate matching of language dialects for translators, and the need for translators specialised in medical terminology.

#### Financial and transport limitations

All participants (including adolescents) spoke of the distress of tight budgets and how this can compromise wellbeing. One stakeholder explained that “*some of the fruits are quite expensive, or the vegetables … [parents] said oh, we buy these biscuits because I can get three for so many.”* A related challenge was navigating transport and the way to appointments.

“*accessing places that they’re not familiar with on public transport because they don’t have the finances to catch a cab, is quite traumatic and quite problematic for them … they’re not comfortable to travel anywhere because they can’t read the street signs, or anything else … they get lost, they arrive late to the appointment which makes the other clinicians angry.”* (Stakeholder)

World Café participants also resonated with the reported finance and transport challenges.

### World Café ideas to facilitate better access to health Services for Refugee Youth

All World Café implementation ideas were categorised as a high or innovative priority according to the PRECEDE-PROCEED matrix; no “low priority” or “no priority” ideas were identified. General themes included ways to improve outreach such as setting-up health clinics or health visitations within schools, English language Centres, playgroups, GP clinics, or at cultural art events. Encouraging “soft entry” into health services via family outings or social conversation groups was also suggested. Novel transport ideas and weekend clinics were offered as a way to encourage health service accessibility. Ideas to improve health literacy included harnessing the media via bilingual discussion of health concepts on the radio and enlisting a culturally-relevant celebrity to promote the importance of early childhood services and play based learning. Interagency collaboration and real-time mapping of families’ navigation through the health system was emphasised, as was the importance of extended case management support and healthcare provider training.

## Discussion

Our study was unique in that we explored refugee children’s developmental health strengths and needs as they related to the key transitions to primary and secondary school. We interviewed refugee parents, their adolescent children, and service providers. This triangulation of perspectives and cross-developmental approach was a significant strength of the study. Not only did it identify common themes - such as the importance of mental health support, strong family systems, English language competence, and social connectedness opportunities for adolescents - it also identified critical divergent themes. This was perhaps most strongly illustrated in participants’ perceptions of services. Refugee families in the current study reported gratitude for services and optimism about the quality of healthcare they received. This contrasted with stakeholder perspectives, which were far less positive about services for refugee families. Stakeholders queried that families *“don’t know what they don’t know”* and that a sense of gratitude creates a barrier to assertive requests for quality healthcare. However, satisfaction with services is a familiar report in the refugee literature. Refugee adults have reported positive experiences with their GP, initial health assessments, midwife, critical moments at hospital [23, 40–46], and relevant to the study aim, child care visits and child health service provision [7, 47]. Where dissatisfaction has been expressed, it is in relation to feelings of discrimination, the attitude of staff manning the health service, and feeling rushed or not listened to sufficiently [24, 47].

The refugee families’ gratitude in the current study possibly reflects the related emphasis that refugee participants placed on personal resilience. Parents’ perceived themselves as resilient, self-sufficient, and hopeful, whereas stakeholders and service providers spoke more to concerns about instability, lack of routine and permissive parenting in families. This has implications for enhancing the perception of refugees’ strengths by service providers, and the transmission of that to families in an empowering way. Indeed, such emphasis encourages a strengths-based model of care that can negotiate a parent versus practitioner led engagement with child health services, at the same time acknowledging the concerns raised by World Café participants that parents’ fear of “losing” their child may encourage delays in health-seeking. The current study extended this fear to figuratively “losing” one’s adolescent child to negative socialisation influences of their resettled country. At a practical level, this is consistent with the World Café suggestion to encourage “soft entry” into health services via social groups, and the recruitment of culturally-relevant celebrities to advocate underutilised health services.

Somewhat contrary to World Café participants’ expectations - as well the literature - refugee families in the current study were largely aware of available health services [7, 23, 42, 48]. The current study was conducted in an area with an established population of Arabic-speaking migrants and refugees, including GPs and health professionals who are Arabic-speaking; thus, the greater awareness could reflect parents’ reports of utilising the longstanding community as a go-to resource about health services, or perhaps an increased likelihood of health material in Arabic language. It might also be a promising sign that systems are improving. For example, in a recent British report, refugee parents were aware of a wide range of services, professionals and community initiatives and resources that could help to keep their children healthy [47]. Nonetheless, the current study identified that a key gap was lack of awareness of (free) early childhood services for children aged 0–5 years. Stakeholders endorsed poor uptake of this resource despite good evidence that early childhood interventions can be highly effective in promoting equity in child health and development [49]. Given the high uptake rates of on-arrival health screens for refugees, linking preschool families with a local playgroup or early childhood service as part of this screen could be one means by which to target this gap in health access (at least amongst refugee families eligible for the health screen).

As in other studies [50], the current findings identified that a central trusted person or caseworker may be critical in assisting families to navigate a complex health system. A new dimension was added of reconciling the disconnect between families’ and practitioners’ priorities, and being based in neutral, trusted settings, such as the GP practice for pre-schoolers and the school for adolescents. This supports investing in resourcing GPs to be culturally congruent and offering the preferred model of school-based mental health services. The continuity of a go-to case manager moreover lends itself to the practice of trauma-informed care in the sense that it promotes the development of trust between families and services. Utilising the school for broader wellbeing support might also assist with parents’ aspirations for their adolescent child to connect with others and pursue goals and meaningful hobbies.

Certainly, parents were insightful as to the needs for their adolescent children, recognising the importance of a prosocial peer group, play, socialisation, sports, hobbies and the pursuit of “joy”. The unmet adolescent need for social activities and belonging, identified by stakeholders also, echoes the refugee literature on the importance of peer relationships for this age group in terms of being a protective factor in mental health [51–53]. It becomes more pressing in the context of research that refugee youth are vulnerable to negative peer experiences [52, 54, 55]. This emphasises a responsibility for better provision of social opportunities for refugee youth. Sports-for-development community programs are one possible avenue [56]. Owning a smartphone was also noted as important for adolescents, and adolescents’ themselves suggested the idea of an online resource by which to facilitate social integration. The speed with which youth are adopting social media, as well as literature citing its role in facilitating the social participation and cohesion of ethnic minority and refugee older adolescents, suggests that, managed safely, social media might provide an engaging platform through which to maintain connectivity alongside or in-between these face-to-face opportunities [57, 58].

Whilst communication in English as a barrier is well known, parents clearly distinguished between written and spoken English, and identified the health service communication through letters as problematic, with *“throwing the [letters] out”*. This has obvious implications for translation of written material to be readily available to health staff. The study finding that inadequate interpreter provision, and financial and transport challenges hinder health care engagement for refugees is also common in the literature [2, 9, 18, 20–25]; and supported by evidence that components associated with improved access to primary healthcare for refugees includes the use of bilingual staff and interpreters, translated material, free transport to and from appointments, and no or low-cost outreach services [50].

Adequate language concordance has been associated with higher reporting of past traumatic events and psychological symptoms, and more referrals to psychological care [59]. In this way, English language competency may have weighted implications for supporting the mental health of refugee children/youth. Contrary to previous research citing mental health stigma as a significant barrier to refugee youth accessing psychological support [22], refugee families in the current study normalised mental health concerns and agreed on the importance of supporting mental health needs, whilst also acknowledging that stigma existed. Stakeholders were more guarded in this perception of how mental health conditions were accepted by refugee families.

### Strengths and limitations of this study

An asset of the study was that refugee families were able to be interviewed in their native language. This diverges from most qualitative studies that often require English language proficiency, thus recruiting a biased, potentially more acculturated, sample of refugees. However, the Arabic focus groups were translated by the bilingual research team rather than accredited professionals. This was due to translator non-availability and/or prohibitively expensive fees. Moreover, the inclusion of only Arabic-speaking parents limits generalisability of the study findings to other refugee-background communities. Another study limitation was that despite efforts to utilise accessible locations and child-minding services, the sample size of pre-schooler parents was small (independent of thematic saturation). This could be indicative of greater barriers associated with parents of this age group, and why greater representation of this age group would be especially beneficial in future studies.

## Conclusion

Refugee children and youth have as much right to come to school healthy and ready to learn as non-refugee children. The methodology used to conduct this study, as well as its findings, emphasise a culturally-tailored approach that considers resettlement stressors and the family’s priorities in a way that is strengths based and resilience building. Indeed, whilst newly arriving refugee families face challenges in accessing appropriate health and support services, they also have strengths that enable them to optimise their children’s wellbeing. A central case manager may be critical to this approach, in addition to embedding models of care within different services or pathways that are valued by each age group, namely GP services for pre-schoolers and schools for adolescents. The World Café method represented a creative conversational process for sharing this knowledge, and began the next steps of operationalising the implications and contextualising them within what matters in the real world.

## Additional file

Additional file 1: Focus Group Guide Focus group vignettes and questions. (DOCX 31 kb)

## Data Availability

The data that support the findings of this study are available from the corresponding author upon reasonable request.
