# Supplementary material for: Optimising refugee children’s health/wellbeing in preparation for primary and secondary school: a qualitative inquiry
Source: BMC Public Health. 2019 Jun 27;19:812. doi: 10.1186/s12889-019-7183-5 (PMC6595577; doi:10.1186/s12889-019-7183-5)
Supplement: Supplementary file 1 — Focus Group Guide Focus group vignettes and questions. (DOCX 31 kb) [file 12889_2019_7183_MOESM1_ESM.docx]

**Focus Group Guide**

**PRESCHOOL GROUP**

***Vignette 1 (Pre-school age child with typical development)***

Parvez is a 4 ½ -year-old boy from Iraq- Kurdish background. Parvez came with his parents and three older sisters to Sydney, two years ago. His father is 60 years old and has many health problems; his mother is 42 years and also has health problems including feeling very sad. Parvez had a normal birth, he breast fed well till two years of age. His mother worries that he doesn’t eat well and she has a hard time trying to get him to brush his teeth. She worries that he gets colds all the time. Parvez is a lovely looking boy. He loves to play with other children, he loves to climb, and he also loves to draw. Parvez has picked up English watching TV at home! His mother takes him to the park where he regularly interacts with the other children, often playing chasings and talking with the other children. He is very kind to the younger children. Parvez will attend school next year and his mother is thinking of putting him in a preschool soon. That will give him three months in the preschool before he starts school. She is wondering if this will be the best thing for him. She worries that he might get sick from other children.

*Parent Questions*

What might Parvez need as a young boy growing up in Australia?

Describe some hopes that Parvez’s parents might have for him?

Describe the family’s and Parvez’s strengths.

How important is it for children to attend preschool?

What age do you think it is important for children to attend preschool?

Describe other services that the family could access for Parvez?

Describe what Parvez and/or his family might need to be prepared for preschool (or school) –

What things might hinder their access to these services or supports – how could the barriers be addressed or managed?

What things might encourage or help Parvez or his parents to access services?

Describe anything else that you think Parvez might need?

*Stakeholder Questions*

How does this story relate to the families that you see within your service?

Describe some of the family’s strengths?

Describe what Parvez and/or his family might need to be prepared for preschool (or school)

Describe what else Parvez might need as a young boy growing up in Australia, or other service that the mother could access for Parvez.

Describe what you might do if this family/child presented at your service?

Describe what might help or hinder their access to these services or supports – how could the barriers be addressed or managed?

Describe what you might like to provide to the child/family that is beyond the service capabilities & your reasons as to why you do not provide that service or cannot access it for the family?

***Vignette 2 - Pre-school age child with developmental concerns***

Azeeza is a four-year-old girl from Iraq. She was diagnosed with Down syndrome, shortly after she was born in Baghdad. This is because of how she looked. She was floppy, had difficulty with breastfeeding and doctors picked up a problem with her heart early. Azeeza has arrived recently in Fairfield with her mother. She has two older brothers who have a muscle weakness problem (they are in wheelchairs) and one older sister. Azeeza’s father died. Azeeza speaks very few words in Arabic or in English. She is slow with her development in every way. Azeeza also needs hearing aids but they are very expensive. Her mother worries that if Azeeza goes to preschool, the hearing aids might get damaged. Azeeza’s mother has been struggling with trying to sort out services for the older brothers. She has not yet worked out what to do with Azeeza’s problems.

*Parent Questions*

Where do you think Azeeza’s mother could or should go for help?

Describe some hopes that Azeeza’s parents might have for her?

Describe the family’s and Azeeza’s strengths.

<How important is it for children to attend preschool?>

<What age do you think it is important for children to attend preschool?>

Describe other services that the family could access for Azeeza?

What would be available in your home country?

Describe what Azeeza and/or his family might need to be prepared for preschool (or school), or as a young girl growing up in Australia more generally?

What things might hinder their access to these services or supports – how could the barriers be addressed or managed?

What things might encourage or help Azeeza or his parents to access services?

Describe anything else that you think Azeeza might need?

*Stakeholder Questions*

How does this story relate to the families that you see within your service?

Where do you think Azeeza’s mother should go for help? What sort of support should Azeeza and her mother get?

Describe what you might do if this family/child presented at your service?

Describe what you might like to provide to the child/family that is beyond the service capabilities & your reasons as to why you do not provide that service or cannot access it for the family?

Describe some of the family’s strengths?

Describe what Azeeza and/or his family might need to be prepared for preschool (or school)

Describe what else Azeeza might need as a young girl growing up in Australia, or other services that the mother could access for Azeeza.

Describe what might help or hinder their access to these services or supports – how could the barriers be addressed or managed?

**ADOLESCENT GROUP**

**Vignette 1 – Adolescent child with typical development**

Zahra is a 12 year old girl. She is a twin that was born healthily in Syria. At a young age she was exposed to a bomb explosion that killed her twin sibling and broke her left leg. According to mum, her leg has been reviewed by her GP here in Australia and showed that it didn’t heal very well. Zahra sometimes gets toothache. Dad experiences low mood and his eyesight is getting worse. The family moved to Australia few years ago. While mum has limited English, the children speak English well. Zahra is about to go into Year 7 of high school. She enjoys school and recently sat her NAPLAN exam. Zahra is described by her teachers as a delightful girl, and is on the student advisory committee. She is popular at school and has lots of friends. Zahra has started to show the first signs of puberty in the last few months.

*Parent Questions*

Describe some hopes that Zahra’s parents might have for her?

What might Zahra need as adolescent girl just entering high school?

What things might hinder their access to these services or supports – how could the barriers be addressed or managed?

What things might encourage or help Zahra or her parents to access services?

Describe other services or things that the family might need for Zahra?

Describe the family’s and Zahra’s strengths.

*Child Questions*

What are your thoughts about Zahra? What is good for Zahra here <think home, at school, or in her social life>?

What is not so good for Zahra here <think home, at school, or in her social life>?

How do you think Zahra and her family could be helped?

Describe anything that you think Zahra might need, especially as she is about to go into high school?

What things might stop or make it difficult for Zahra to get things or the support she might need?

What things might encourage or help Zahra to get things or the support she might need?

*Stakeholder Questions*

How does this story relate to the families that you see within your service?

Describe some of Zahra’s strengths?

Describe what Zahra and/or her family might need to be prepared for high school or as a young adolescent girl growing up in Australia?

Describe what you might do if this family/child presented at your service?

Describe what might help or hinder their access to these services or supports – how could the barriers be addressed or managed?

Describe what you might like to provide to the child/family that is beyond the service capabilities & your reasons as to why you do not provide that service or cannot access it for the family?

Tell me about other services that the family could access for her child?

**Vignette 2 – Adolescent with social and emotional problems**

Emad is a 13 year old boy. His family are from Syria. They left Syria 4 years ago and moved to Jordan where they lived until they arrived in Australia 4 months ago. His dad was badly injured while in a nearby market when a bomb exploded. His mother hopes that he will have a good education and be able to work and help support his younger siblings. He stills gets flashbacks of the day when the bomb exploded in the market andhe thought his dad might be dead. He cannot concentrate for too long and he gets extremely anxious when meeting people he doesn’t know and is worried that he will let his mum down. The family has heard from another refugee family that their son is being bullied at school. His mum is worried but does not know how to help if this happens to her son. The teacher described Emad as having lots of potential but his behaviour can be challenging. He will sometimes ‘act young’ and then at other times be very mature. He is defiant and often will not follow his teacher’s instructions. The teacher reports that he has had significant gaps in his education in the past, but is being supported through extra classes to focus on English.

*Parent Questions*

Where do you think Emad’s mother could or should go for help?

Describe some hopes that Emad’s parents might have for him?

Describe the family’s and Emad’s strengths.

Describe what Emad and/or his family might need to be prepared for high school, or as an adolescent boy growing up in Australia more generally?

Are there any services that the mother could access for Emad?

What would be available in your home country?

What things might stop or make it difficult for Emad to get support?

What things might encourage or help Emad to get support?

*Child Questions*

What do you think is going on for Emad here? What is good for Emad here; what is not so good?

What do you think Emad should do?

Describe anything that you think Emad might need, especially as he is about to go into high school?

How do you think Emad and his mother could be helped?

What would be available in your home country?

What things might stop or make it difficult for Emad to get things or the support he might need?

What things might encourage or help Emad to get things or the support he might need?

*Stakeholder Questions*

How does this story relate to the families that you see within your service?

Where do you think Emad’s mother should go for help? What sort of support should Emad and his mother get?

Describe what you might do if this family/child presented at your service.

Describe what you might like to provide to the child/family that is beyond the service capabilities & your reasons as to why you do not provide that service or cannot access it for the family.

Describe some of the family’s strengths.

Describe what Emad and/or his family might need to be prepared for high school or as an adolescent boy growing up in Australia.

Describe what might help or hinder their access to these services or supports – how could the barriers be addressed or managed?
